# Detection of *CYP2C19* Genetic Variants in Malaysian Orang Asli from Massively Parallel Sequencing Data

**DOI:** 10.1371/journal.pone.0164169

**Published:** 2016-10-31

**Authors:** Geik Yong Ang, Choo Yee Yu, Vinothini Subramaniam, Mohd Ikhmal Hanif Abdul Khalid, Tuan Azlin Tuan Abdu Aziz, Richard Johari James, Aminuddin Ahmad, Thuhairah Abdul Rahman, Fadzilah Mohd Nor, Adzrool Idzwan Ismail, Kamarudzaman Md. Isa, Hood Salleh, Lay Kek Teh, Mohd Zaki Salleh

**Affiliations:** 1 Integrative Pharmacogenomics Institute (iPROMISE), Universiti Teknologi MARA Selangor (UiTM), Puncak Alam, Selangor, Malaysia; 2 Faculty of Pharmacy, Universiti Teknologi MARA Selangor (UiTM), Puncak Alam, Selangor, Malaysia; 3 Faculty of Medicine, Universiti Teknologi MARA Selangor (UiTM), Sungai Buloh, Selangor, Malaysia; 4 College of Arts and Sciences, Universiti Utara Malaysia (UUM), Sintok, Kedah, Malaysia; 5 Faculty of Communication and Media, University Selangor, Shah Alam, Selangor, Malaysia; 6 Institut Alam Sekitar dan Pembangunan (LESTARI), Universiti Kebangsaan Malaysia (UKM), Bangi, Selangor, Malaysia; Pennsylvania State University College of Medicine, UNITED STATES

## Abstract

The human cytochrome P450 (CYP) is a superfamily of enzymes that have been a focus in research for decades due to their prominent role in drug metabolism. CYP2C is one of the major subfamilies which metabolize more than 10% of all clinically used drugs. In the context of CYP2C19, several key genetic variations that alter the enzyme’s activity have been identified and catalogued in the CYP allele nomenclature database. In this study, we investigated the presence of well-established variants as well as novel polymorphisms in the *CYP2C19* gene of 62 Orang Asli from the Peninsular Malaysia. A total of 449 genetic variants were detected including 70 novel polymorphisms; 417 SNPs were located in introns, 23 in upstream, 7 in exons, and 2 in downstream regions. Five alleles and seven genotypes were inferred based on the polymorphisms that were found. Null alleles that were observed include CYP2C19*3 (6.5%), *2 (5.7%) and *35 (2.4%) whereas allele with increased function *17 was detected at a frequency of 4.8%. The normal metabolizer genotype was the most predominant (66.1%), followed by intermediate metabolizer (19.4%), rapid metabolizer (9.7%) and poor metabolizer (4.8%) genotypes. Findings from this study provide further insights into the *CYP2C19* genetic profile of the Orang Asli as previously unreported variant alleles were detected through the use of massively parallel sequencing technology platform. The systematic and comprehensive analysis of *CYP2C19* will allow uncharacterized variants that are present in the Orang Asli to be included in the genotyping panel in the future.

## Introduction

Genetic factors are known to contribute towards interindividual variation in drug disposition particularly mutations that occur in genes that encode for drug-metabolizing cytochrome P450 (CYP) enzymes [1]. CYP is a superfamily of heme-containing monooxygenases; these diverse enzymes are further classified into 18 families and 43 subfamilies based on amino acid homology [2, 3]. CYP enzymes play a crucial role in the metabolism of drugs as well as other xenobiotic and endogenous compounds by catalyzing a variety of reactions especially oxidative reactions. A total of 115 CYP genes comprising of 57 active and 58 pseudogenes were discovered following the release of the complete human genome sequence but 90% of drug metabolism activities are attributed to a small number of core enzymes including CYP1A2, CYP3A4, CYP2B6, CYP2C9, CYP2C19, CYP2D6 and CYP2E1 [4, 5]. Genetic variations in CYP enzymes are extensively studied because significant alteration in the enzyme activity poses drug toxicity risk and may lead to therapeutic failure. In Malaysia, 13001 cases of adverse drug reaction were reported to the National Pharmaceutical Control Bureau in 2014 and the number of cases had doubled compared to that of 2009 (5850 cases) [6]. Therefore, preemptive genotyping of genes that are known to be involved in the metabolism of a particular drug may minimize the risk of adverse drug reaction by allowing drug selection and dosage adjustment to be carried out based on the patient’s inferred phenotype before the initiation of treatment [7].

CYP2C19 is similar to other major CYP enzymes whereby metabolism of its substrates is influenced by a number of factors such as variations in allelic composition and the level of hepatic expression [8]. CYP2C19 is one of the four functional members in the CYP2C subfamily with the other three being CYP2C8, CYP2C9 and CYP2C18. *CYP2C19* is located on chromosome 10q23.33; the gene is 90209 bp in length and its 1473-bp coding region consists of nine exons. CYP2C19 is predominantly expressed in the liver but lower levels of the enzyme can also be found in the small intestine where it contributes to the first-pass metabolism of its substrate drugs [9, 10]. CYP2C19 is known to be involved in the biotransformation of at least 10% of commonly prescribed drugs which include the antiplatelet pro-drug clopidogrel, proton pump inhibitors (e.g. omeprazole and lansoprazole), tricyclic antidepressants (TCAs) (e.g. imipramine and clomipramine), selective serotonin-reuptake inhibitors (SSRIs) (e.g. citalopram and venlafaxine), anticonvulsants (e.g. diazepam and phenytoin) and anti-infectives (e.g. proguanil and voriconazole) [8, 11]. There are 35 variants (*) alleles of *CYP2C19* that have been defined by the CYP allele nomenclature committee to date (http://www.cypalleles.ki.se/cyp2c19.htm) but two of the most common variant alleles are CYP2C19*2 and *3 which result in no functional CYP2C19 enzyme [12–14].

Variations in the prevalence of CYP2C19*2 and *3 have been observed in populations of different ethnicities and geographical origins with reported frequencies ranging from 0.3% to 54.9% (15 Besides CYP2C19*2 and *3, other null alleles or alleles with decreased enzyme activity include *4, *5, *6 and *8 which are rare variants that generally occur at frequencies less than 1% in various populations including Caucasians, Asians and Africans [15]. CYP2C19*17 is the only allele with increased function that has been described so far and it is most prevalent among Caucasians (European and North American) [15]. The increase in CYP2C19 expression and activity were attributed to the creation of a consensus binding site for GATA transcription factor family due a -806C>T mutation (rs12248560) in the promoter region [16]. From the clinical perspective, an individual can be assigned a predicted phenotype of ultra-rapid metabolizer (UM), rapid metabolizer (RM), normal metabolizer (NM), intermediate metabolizer (IM) or poor metabolizer (PM) based upon the diplotypes of the above allelic variants [17]. Individuals with two alleles of increased function or more than two alleles with normal function are classified as UMs while combinations of alleles with normal function and increased function are seen in RMs. Combinations of alleles with normal function and decreased function occur in NMs whereas combinations of alleles with normal function, decreased function and/or no function occur in IMs. Individuals with combination of alleles with no function and/or decreased function are PMs as they have little to no CYP2C19 enzyme activity.

Continuous efforts in characterizing and identifying key genetic variations that are associated with this highly polymorphic pharmacogene as well as the development of dosing guidelines for clopidogrel, selective serotonin-reuptake inhibitors and tricyclic antidepressants based on *CYP2C19* genotype by the Clinical Pharmacogenetics Implementation Consortium (CPIC) [15, 18, 19] have made preemptive genotyping a viable strategy for personalized medicine. Hence, prior knowledge of the genetic variants that are present in a population would facilitate the revision and optimization of existing pharmacotherapy practices and policies. In Malaysia, the allele frequencies of *CYP2C19* in Malay, Chinese and Indians, which represent the three major ethnic groups in this country, as well as aboriginal populations such as the Orang Asli in the Peninsular (West Malaysia) and Iban in the East Malaysia were studied by targeting specific variants [20–22] and hence, the frequencies of undetected alleles remained unknown. In the present study, high-throughput sequencing was performed to investigate the genetic diversity of *CYP2C19* in the Orang Asli from six sub-tribes. The comprehensive analysis of the whole gene will provide insights into variants that would have been missed by selective amplification and contribute to the pharmacogenomics knowledge base of the minority Orang Asli group.

## Materials and Methods

### Subjects

Sixty-two healthy and unrelated participants from six different sub-tribes of Orang Asli were recruited in this study. Sub-tribes that are located in the northern region of the Peninsular Malaysia are Bateq (*n* = 9; Gua Musang, Kelantan), Lanoh (*n* = 10; Lenggong, Perak) and Kensiu (*n* = 10; Baling, Kedah) while those in the central region are Che Wong (*n* = 13; Kuala Gandah, Pahang) and Semai (*n* = 14; Kuala Lipis, Pahang) whereas the Kanaq (*n* = 6) settlement lies in the southern region of Kota Tinggi, Johor. The demographic data of the Orang Asli for each sub-tribe is presented in Table 1. All the participants were interviewed to ascertain that there were no mixed lineages from intermarriage for at least three generations. The research objectives were explained to all the participant and informed consent was obtained and documented through the use of a written consent form prior to blood sample collection. The study protocol and consent procedure were approved by the Research Ethics Committees of Universiti Teknologi MARA [600-RMI (5/1/6/01)] and Malaysia Orang Asli Development Department [JAKOA.PP.30.052 Jld 5(62)].

**Table 1:** Demographic data of the Orang Asli.

Tribes	Age (years)		Gender	
	Mean	Range	Male	Female
Negritos				
Bateq	32.8	20–45	8	1
Kensiu	42.2	18–59	5	5
Lanoh	40.0	17–67	5	5
Senoi				
Che Wong	33.3	18–70	5	8
Semai	34.6	19–52	5	9
Proto-Malay				
Kanaq	35.4	23–52	1	5

### DNA extraction and high-throughput sequencing

Extraction of genomic DNA from blood samples were performed using Wizard Genomic DNA Purification Kit (Promega, Wisconsin, USA). The purity and quantity of the extracted genomic DNA was determined using Quantus Fluorometer (Promega) and NanoDrop 2000 UV-Vis spectrophotometer (Thermo Scientific, MA, USA). Sequencing libraries with an insert size of 550 bp were then constructed using TruSeq DNA PCR-Free Library Preparation Kit (Illumina, San Diego, CA, USA) and pair-end sequencing was carried out using Genome Analyzer (Illumina) (*n* = 24; 30 × average coverage) and MiSeq instrument (Illumina) (*n* = 38; 2–15 × average coverage). Quality of the raw sequencing reads was checked with FastQC [23] and trimmed with SolexaQA version 2.5 [24] before the data were processed according to the Genome Analysis Toolkit (GATK) Best Practices recommended workflows [25]. Briefly, the reads were aligned to reference human genome GRCh37/hg19 using BWA version 0.6.1-r104 [26] and duplicates were marked and removed using Picard version 1.119 (http://broadinstitute.github.io/picard/). Indel realignment and base quality score recalibration was conducted using GATK version 2.5–2.

### Variant discovery and data analysis

Genetic variants in *CYP2C19* of each individual were called using GATK HaplotypeCaller and the generated output was manually inspected by comparison to the wild type nucleotide sequence of *CYP2C19* (NG_008384.2). Each genetic variant was assigned with a reference cluster ID (rs number) from NCBI Database of Single Nucleotide Polymorphisms (dbSNP) build 146 [27], when available. Alleles were assigned based on the major SNPs/alterations that were specified by the human CYP allele nomenclature committee as being responsible for the phenotype of the corresponding allele. Hardy–Weinberg equilibrium for each SNP and pairwise linkage disequilibrium (LD) between variants with MAF ≥ 0.05 and HWpval (Hardy-Weinberg equilibrium p-value) > 0.001 were analyzed using Haploview 4.2 [28]. The online tools SIFT [29]and PolyPhen-2 [30] were used to predict the functional effect on protein caused by non-synonymous SNP in *CYP2C19* coding regions. An amino acid substitution is predicted to be damaging if the SIFT score is equal or below 0.05 whereas a score above 0.05 indicates that the substitution is tolerated. PolyPhen-2 predicts the effect of an amino acid substitution as either benign, possibly damaging or probably damaging with scores ranging from 0–1.

## Results

### Genetic variants

We found 449 *CYP2C19* genetic variants in the Orang Asli genomes and 70 of these polymorphisms have not been reported in dbSNP build 146. There were only seven exonic SNPs as opposed to 417 intronic SNPs and 25 SNPs in the upstream (*n* = 23) and downstream (*n* = 2) regions. Intron 5 possessed the largest number of SNPs (*n* = 205) including novel variants followed by intron 6 with 86 SNPs including 11 novel variants. The remaining 15 novel polymorphisms were located in the upstream region (*n* = 3), intron 1 (*n* = 1), intron 3 (*n* = 3), intron 7 (*n* = 5) and intron 8 (*n* = 3). Among the seven SNPs in exonic regions, four were synonymous and three were non-synonymous. Minor allele frequencies of the genetic variants that were found in this study and the Hardy–Weinberg equilibrium test result are shown in S1 Table.

### Allele and genotype frequency

A total of five alleles and seven genotypes were inferred based on the genetic variants of *CYP2C19* that were detected in this study. The normal function CYP2C19*1 was the most common allele in the Orang Asli which occurred at a frequency of 80.7% whereas the combined frequency of no function alleles (CYP2C19*2, *3 and *35) was 14.5% and increased function allele (*17) was 4.8% (Table 2). The allele frequencies of CYP2C19*1 was over 80% in all sub-tribes except for Lanoh (70%) and Che Wong (69.2%). The frequencies of variant alleles CYP2C19*2 and *3 were comparable although *3 (6.5%) was found at a slightly higher frequency than *2 (5.7%). The allele with the lowest frequency was CYP2C19*35 (2.4%). CYP2C19*2 was detected in Lanoh (15%), Semai (7.1%), Kensiu (5%) and Che Wong (3.9%) but not the Bateq and Kanaq sub-tribes. On the other hand, CYP2C19*3 was only found in two sub-tribes and the frequency of *3 was much higher in Che Wong (26.9%) than Kensiu (5%). In the Kanaq, CYP2C19*35 (16.7%) was the only variant allele that was identified. CYP2C19*17 was observed in Lanoh (15%), Bateq (11.1%) and Kensiu (5%).

**Table 2:** Allele frequencies of *CYP2C19* in the Orang Asli.

Allele	Phenotype	Allele frequency (%)						
		Negritos			Senoi		Proto-Malays	All sub-tribes (*n* = 62)
		Bateq (*n* = 9)	Kensiu (*n* = 10)	Lanoh (*n* = 10)	Che Wong (*n* = 13)	Semai (*n* = 14)	Kanaq (*n* = 6)	
*1	Normal function	83.3	85.0	70.0	69.2	92.9	83.3	80.7
*2	No function	0	5.0	15.0	3.9	7.1	0	5.7
*3	No function	0	5.0	0	26.9	0	0	6.5
*17	Increased function	11.1	5.0	15.0	0	0	0	4.8
*35	No function	5.6	0	0	0	0	16.7	2.4

The NM genotype was predominant in the Orang Asli as 66.1% carried CYP2C19*1/*1 (Table 3). Overall, the frequency of CYP2C19*1/*1 was over 60% in all sub-tribes except Lanoh (40%) whereas the combined frequency of IM genotypes in the Orang Asli, which comprised of CYP2C19*1/*2, *1/*3 and *1/*35, was only 19.4%. PM genotypes accounted for 4.8% and interestingly, Che Wong was the only sub-tribe in which CYP2C19*2/*3 and *2/*3 were detected. Individuals with the RM genotype were only detected in the Bateq, Kensiu and Lanoh with frequencies ranging from 10% to 30%. The allele frequencies of *CYP2C19* in the Orang Asli were compared with other populations as shown in Table 4.

**Table 3:** *CYP2C19* genotype frequencies in the Orang Asli.

Genotype	Phenotype	Allele frequency (%)						
		Negritos			Senoi		Proto-Malays	All sub-tribes (*n* = 62)
		Bateq (*n* = 9)	Kensiu (*n* = 10)	Lanoh (*n* = 10)	Che Wong (*n* = 13)	Semai (*n* = 14)	Kanaq (*n* = 6)	
*1/*1	Normal metabolizer	66.7	70.0	40.0	61.5	85.7	66.7	66.1
*1/*2	Intermediate metabolizer	0	10.0	30.0	0	14.3	0	9.7
*1/*3	Intermediate metabolizer	0	10.0	0	15.4	0	0	4.8
*1/*17	Rapid metabolizer	22.2	10.0	30.0	0	0	0	9.7
*1/*35	Intermediate metabolizer	11.1	0	0	0	0	33.3	4.8
*2/*3	Poor metabolizer	0	0	0	7.7	0	0	1.6
*3/*3	Poor metabolizer	0	0	0	15.4	0	0	3.2

**Table 4:** Comparison of *CYP2C19* allele frequencies between the Orang Asli and various populations.

Population	Allele frequency (%)					Reference
	*1	*2	*3	*17	*35	
**Malaysian**						
Orang Asli	80.7	5.7	6.5	4.8	2.4	This study
Orang Asli	84.1	15.9	-	-	-	[21]
Chinese	58.8–67.4	28.9–30.9	3.7–10.3	-	-	[20, 21]
Indian	62.5–67.3	31.0–37.5	0–1.75	-	-	[20, 21]
Malay	72.2–77.5	20.1–23.2	2.4–4.6	-	-	[20, 21]
**Other major race/ethnic groups**						
African	36.4	14.2	0.8	15.1	-	[15]
African American	58.1	18.3	0.3	19.4	-	[15]
Caucasian (European and North American	62.1	14.6	0.6	21.5	-	[15]
Middle Eastern	84.2	13.2	2.6	-	-	[15]
East Asian	58.0	29.0	8.5	1.6	-	[15]
South/ Central Asian	47.4	34.4	1.7	16.5	-	[15]
Americas	69.0	13.1	0.3	16.3	-	[15]
Oceanian	28.6	54.9	13.9	2.5	-	[15]

### Linkage disequilibrium analysis

**Fig 1:**
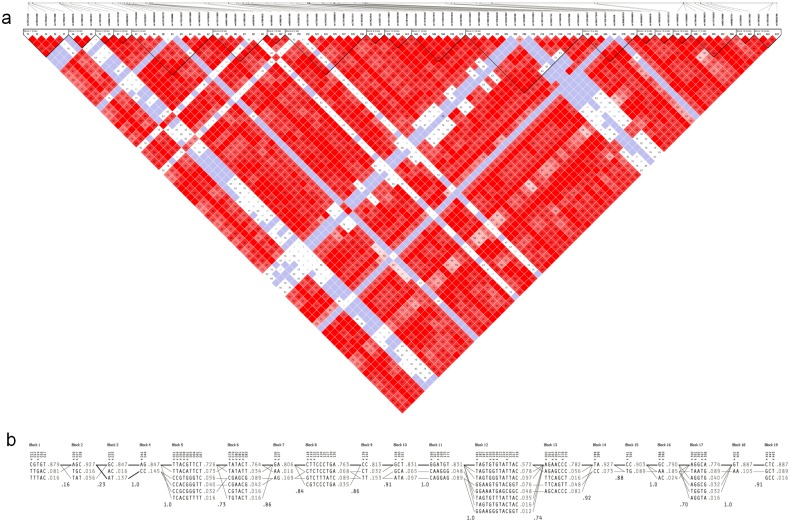
Analysis of haplotype structures at CYP2C19 locus in the Orang Asli. (a) Linkage disequilibrium map showing 19 haplotype blocks along with the reference SNP cluster ID (rs). Bright red depicts very strong LD (LOD ≥2; D’ = 1), pink red (LOD ≥2; D’ < 1) and blue (LOD < 2; D’ = 1) for intermediate LD and white for no LD (LOD < 2, D’ < 1) between the pair of SNPs. Numbers in the square were the D’ value multiplied by 100. (b) Haplotype structures of CYP2C19 with tagged SNP indicated by an inverted triangle. Frequency of each haplotype is shown at the edge and the multi-allelic D’ value between each block is shown beneath. The most common crossings between haplotypes are indicated by thick lines whereas less common crossings are indicated by thinner lines.

Regions of low recombination in *CYP2C19* locus of the Orang Asli were analyzed using Haploview and 19 blocks of SNPs in linkage disequilibrium were identified (Fig 1). Complete LD (D' = 1) between markers was observed in a total of 8 blocks. The largest haplotype block with 13 markers (block 12) spanned across 5 kb and the second largest block (block 13) with 7 markers spanned across 4 kb. Both blocks exhibited an intermediate level of LD and were located in the intron 5 region. The multi-allelic D' values, which correspond to the level of recombination between two blocks, ranged from 0.16 which indicated a great amount of historical recombination to 1.0 for no evidence of historical recombination.

## Discussion

In the present study, the genetic diversity of *CYP2C19* in the Orang Asli from six different sub-tribes were studied and reported for the first time. The Orang Asli are the indigenous people of Peninsular Malaysia and the estimated population size is only ~180,000 people [31]. The Senoi tribe, which consists of six sub-tribes (Che Wong, Jahut, Mah Meri, Semoq Beri, Semai and Temiar) formed the largest group of Orang Asli with ~ 98,000 people followed by the Proto-Malays with ~75,000 people from 6 sub-tribes (Kanaq, Kuala, Seletar, Jakun, Semelai and Temuan) and the smallest group are those that expressed the negrito phenotypes (Bateq, Jahai, Kensiu, Kintaq, Mendriq and Lanoh) with an estimated number of only 5,000 people [32]. Their settlements are scattered across the Peninsular Malaysia whereby the Negritos and Senoi are generally localized in the northern and central regions while Proto-Malays settlements are found in the central and southern regions [33].

Some of the sub-tribes selected in the present study were identified by the Department of Development of the Orang Asli, Malaysia (http://www.jakoa.gov.my) as having a small population number namely Kanaq (*n* = 65), Kensiu (*n* = 204), Lanoh (*n* = 359), Che Wong (*n* = 417) and Bateq (*n* = 1004). The small population size, limited knowledge of family pedigrees and intermarriages with other ethnic groups were among the factors that contributed to the small number of participants in this study. These substribes are therefore fragile population whose genetic variability are of great interest to scientists that focus on natural selection and aboriginal studies. Overall, data mining from the genome of 62 Orang Asli resulted in the detection of 449 *CYP2C19* genetic variants including 70 novel polymorphisms, five alleles and seven genotypes. Among the three non-synonymous mutations that were detected, 17948G>A (rs4986893) is the hallmark SNP of CYP2C19*3 that results in the production of a non-functional enzyme due to the presence of a premature termination codon at amino acid 212 (Trp212Ter) [13] while Ile331Val substitution caused by 80161A>G (rs3758581) is present in the majority of variant alleles and does not appear to affect the enzyme activity [34]. The 17830C>T (rs61311738) variation in exon 4 which causes Ala173Val substitution did not belong to any defined haplotype in the *CYP2C19* allele nomenclature database (http://www.cypalleles.ki.se/cyp2c19.htm) to date and may potentially be assigned a unique allele designation. The haplotype of this SNP could not be determined in the present study as the MAF was less than 0.05. The change in amino acid at position 173 in CYP2C19 was predicted to be tolerated (0.09) and benign (0.355) based on SIFT and PolyPhen-2 analysis, respectively. However, its function needs to be further assessed *in vitro* or *in vivo* to unequivocally determine the effect of Ala173Val substitution.

The allele with normal function CYP2C19*1 was found at a frequency of 80.7% in our Orang Asli cohort, which is comparable to the findings by Yusoff et al. [21] who reported a frequency of 85.1% for an Orang Asli cohort comprising of 176 subjects. However, this study did not provide tribal information and allele frequencies that were derived from a single sub-tribe may not be representative of the Orang Asli population at large. A total of 11 variant alleles (CYP2C19*2, *3, *4, *5, *6, *10, *11, *12, *13, *14 and *15) were screened by Yusoff et al. [21] using a nested amplification-refractory mutation system-PCR approach but *17 and *35 were not tested which could be the likely factor contributed to an overestimation of the *1 allele. Overall, the *1 allele frequency of the Orang Asli (80.7%) was higher than that of the three major ethnics in Malaysia (58.8–77.5%) (20, 21) and closer to that of Middle Eastern (84.2%) than East Asian (58%), South/Central Asian (47.4%) and Africans (36.4%) [15]. CYP2C19*2 and *3 are the major variant alleles that were detected in our Orang Asli cohort. However, CYP2C19*2 allele was more prevalent in Malaysian Malay, Chinese and Indian (23.2–37.5%) [20, 21] as well as other major race/ethnic groups (13.1–54.9%) [15] when compared to the Orang Asli (5.7%). The frequency of CYP2C19*3 allele in the Orang Asli was higher than Malaysian Indian and Malay (0–4.6%) but in between that of populations from Middle Eastern (2.6%) and East Asian (8.5%).

Besides CYP2C19*2 and *3, we have also detected *35 in the Orang Asli genomes and the allele occurred at a frequency of 2.4%. CYP2C19*35 encodes for a non-functional, truncated protein because nucleic acid substitution from adenine to guanine at position 12662 results in a premature stop codon due to the altered mRNA reading frame [35]. This variant allele was described by Chaudhry et al. in 2015 [35] and has only been found in Blacks with African ancestry to date. In the present study, the finding of CYP2C19*35 in the Bateq sub-tribe may be attributed to their African heritage but the allele was not detected in Kensiu and Lanoh even though they are sub-tribes of Negrito. Of particular note, the finding of CYP2C19*35 in the Proto Malays of Kanaq sub-tribe who were descendants of Mongoloid people indicated that allele is not exclusive to a unique ethnic group. The allele with increased function CYP2C19*17 was also detected for the first time in the Orang Asli population and the allele frequency (4.8%) was higher than that reported by Tiong et al. [22] among 323 patients that were planned for percutaneous coronary intervention in East Malaysia (2%). Nevertheless, CYP2C19*17 was more prevalent among Caucasian, African American, South/Central Asian, Americas and African (15.1–21.5%) [15] when compared to the Orang Asli. In agreement with the findings of Yusoff et al. [21], CYP2C19*4, *5, *6, *10, *11, *12, *13, *14 and *15 were not detected in our Orang Asli cohort as well.

In the present study, NM and IM genotypes were found in all sub-tribes, RM genotype was found in three sub-tribes and PM genotypes were only detected in the Che Wong sub-tribe. Differences in the allele and genotype frequencies of *CYP2C19* between different sub-tribes were observed because the Orang Asli are not a homogeneous group and were proposed to have diverse origins [36]. The finding of PM and RM genotypes in the Orang Asli population indicated that *CYP2C19* genotyping may be performed to detect individuals with these allelic variants. In the case of antiplatelet pro-drug, clopidogrel, metabolism of the drug is impaired in individuals with loss-of-functions *CYP2C19* alleles leading to lower active metabolite exposure [37, 38] and the presence of these alleles in patients with acute coronary syndromes and/or those undergoing percutaneous coronary intervention has been associated with an increase in the rates of cardiovascular events [39, 40]. On the contrary, platelet inhibition and clopidogrel response were observed to be enhanced among UM patients [40, 41] although they were also associated with an increased risk of bleeding complications particularly in patients who are homozgyous for CYP2C19*17 [42]. *CYP2C19* genotypes also affect proton pump inhibitor-based treatment as IM and PM tend to respond better due to the reduction of drug elimination and higher plasma concentration of drug [43] while UM are more likely to experience sub-therapeutic drug exposures due to the increased rates of drug metabolism [44]. TCAs are usually prescribed as therapy for depression as well as to relieve chronic pain such as neuropathic pain but these medications also tend to cause a host of side effects [45]. Variability in tricyclic plasma concentrations has been linked to CYP2C19 metabolizer status and patients may benefit from CYP2C19 genotyping as the identification of variant alleles that have been shown to alter drug clearance or the ratio of parent drug to metabolites would allow physician to adjust the drug dosage or select an alternative drug that are not affected by CYP2C19 [19]. SSRIs are the first-line treatment for major depressive disorders but it has been estimated that 50% of these patients will fail to respond to the initial treatment [46] and hence, SSRIs dosing recommendation based on *CYP2C19* genotype could potentially improve treatment outcome and minimize the occurrence of side effects. The metabolism of several SSRIs such as citalopram, escitalopram, and sertraline have been shown to be under the influence of *CYP2C19* genotype whereby the probability of therapy failure among UM is higher due to the significantly lower exposure of these drugs in UM when compared to NM [47, 48] while side effects have been reported to be more common in CYP2C19 PM than NM [49].

In conclusion, a systematic analysis of the *CYP2C19* genetic variants in our Orang Asli cohort has revealed the presence of variant alleles CYP2C19*17 and *35 in addition to *2 and *3 which have previously been found in the Orang Asli by Yusoff et al. [21]. Alteration of the enzyme activity as a direct result of genetic variations has been shown to potentially have clinical implications and as indicated in CPIC guidelines for clopidogrel, TCAs and SSRIs [15, 18, 19], patients with predicted UM or PM phenotypes may benefit from adjusting drug dosage or an alternative drug choice. Although findings from the present study may not be extrapolated to the Orang Asli population or sub-tribes due to the small sample size, the comprehensive data compiled in this study will serve as a guide for future *CYP2C19* genetic analysis to be conducted with a larger sample size. Profiling of the Orang Asli *CYP2C1*9 provides insights into their genetic make-up and allow future pharmacogenetic test that encompasses all relevant variations that are present in the Malaysia population to be developed so that pharmacotherapy for Malaysians can be further optimized and improved.

## Supporting Information

S1 Table Positions and frequencies of *CYP2C19* genetic variants in Orang Asli. (XLSX)
